# Rapid Analytical Method to Characterize the Freshness of Olive Oils Using Fluorescence Spectroscopy and Chemometric Algorithms

**DOI:** 10.1155/2020/8860161

**Published:** 2020-07-11

**Authors:** Aimen El Orche, Mustapha Bouatia, Mohamed Mbarki

**Affiliations:** ^1^Laboratory of Chemical Processes and Applied Materials, Faculty of Science and Technology, Sultan Moulay Slimane University, Beni-Mellal, Morocco; ^2^Laboratory of Analytical Chemistry & Bromatology, Faculty of Medicine and Pharmacy, Mohammed V University in Rabat, Rabat, Morocco

## Abstract

One of the most important issues in the field of quality assurance of olive oils is the detection of the freshness of olive oil. In this study, 400 nm laser-induced fluorescence spectroscopy was used with supervised and unsupervised multivariate analysis methods to develop a rapid method able to discriminate between freshly produced olive oils and oil that has been stored for a period of time ranging from 12 to 24 months. The fluorescence spectral data were firstly processed by the PCA. This method shows strong discrimination of the three oil classes using the first three components which present 96% of the total variability of the initial data, and then supervised classification models were constructed using the discriminant partial least square regression PLS-DA, support vector machine SVM, and linear discriminant analysis LDA. These methods show a high capacity in the classification of the three classes of olive oil. The validation of these classification models by external samples shows a high capacity of classification of the samples in their class with an accuracy of 100%. This study demonstrated the feasibility of the fluorescence spectroscopy fingerprint (routine technique) for the classification of olive oils according to their freshness and storage time.

## 1. Introduction

Olive oil is an important vegetable oil in the Mediterranean countries; currently, this nutrient is attracting the attention of many consumers around the world, thanks to its nutraceutical and sensory properties and contribution to the protection of the human well-being [1]. These proprieties are especially related to its composition rich in fatty acids especially oleic and linoleic acid [2] and its high level of minor compounds that have bioactive characteristics, principally phenolic compounds and tocopherol [3, 4].

These natural biochemical compounds of virgin olive oil are able to delay the effects of oxidation by deactivating the singular oxygen [5, 6]. The greenish coloration of olive oil is attributed to the chlorophyll pigments formed essentially from chlorophylls and their derivative products [7]. The quantification of these compounds in olive oils is considered to be very important for determining the quality of olive oil because the decrease in chlorophyll levels during storage indicates the presence of oxidation processes that affect the quality of olive oil [4, 8]. Its concentration in olive oil depends on several factors such as geography, edaphic factors, climate, storage conditions, ripening stage, and type of extraction [9–12].

Moreover, these compounds are significantly decreased during the storage of olive oil, although new products appear due to the oxidation process [7, 13]. In many markets, the storage of olive oil can vary between 6 and 24 months so that it causes an alteration in the quality of olive oil.

Nowadays, the authentication of olive oils is still a major problem. Virgin olive oil, due to its high price compared with other edible oils, can be the object of more or less sophisticated fraudulent practices. The most common ones consist of adulterating virgin olives oils with lower-priced oils (seed oils, refined olive oil, or olive pomace oil). These practices have been the subject of numerous studies aimed at combating frauds that disrupt the market and damage the importance of virgin olive oil (VOO) [14–16]. There is also another type of fraud that consists of falsifying the freshness of olive oil and presents to the consumer nonfresh olive oils, that have been stored for a period of time, as freshly produced.

Authentication of the VOO belonging to a designation of origin often constitutes a real analytical challenge. For this reason, a great deal of research has been devoted to answering this authentication problem, in order to develop robust and reliable analytical tools able to retrieve all the information on the quality, safety, and origin of olive oil and other oils [17]. These analytical tools can be classified in two main categories, those based on the analysis of chemical compounds of olive oils, gas chromatography (GC) [18, 19] and high-performance liquid chromatography (HPLC) [19–21], and those based on spectroscopic techniques, such as infrared spectroscopy IR [22–24], ultraviolet-visible spectroscopy (UV-visible) [24, 25], magnetic nuclear resonance (MNR) [26], and fluorescence spectroscopy [27, 28], which have been used for adulteration detection, origin geographic determination, variety determination, and examination of the oxidative stability of olive oils.

The HPLC and GC, as reference methods, are generally time-consuming, sometimes require the use of expensive and polluting reagents, and are only performed by qualified operators. Moreover, these methods are not sufficiently efficient to cover the growing demand for an analytical procedure that requires several hours. The use of spectroscopic methods, such as fluorescence combined with chemometric tools, makes possible the realization of these evaluations in a few times without using reagents.

Fluorescence spectroscopy is a specific, nondestructive and rapid analytical tool for the food authentication study [29]. It provides information on the presence of fluorescent molecules and the fluorescence properties of fluorophores. Recently, the application of fluorescence spectroscopy in combination with chemometric tools to evaluate the quality of olive oil has been increased in the majority of research papers [30], because the obtained fluorescence signal corresponds to specific fluorophores such as vitamin E and chlorophyll [31], after having defined the excitation or emission wavelength [32].

This analytical method is combined usually with chemometric approaches using multivariate data processing to extract information from spectroscopic data. Chemometric methods can be supervised or unsupervised. The applications of fluorescence spectroscopy coupled to multivariate analysis with more or less complex preprocessing and sometimes with different excitation and emission wavelengths have been developed by several authors. However, the obtained results in different studies are difficult to compare since the performance criteria and reference value ranges are different.

The present study aims to develop a rapid method based on fluorescence spectroscopy coupled to supervised and unsupervised chemometric algorithms to determine the membership of virgin olive oil in a group of olive oils. The first aim of the work is to know if these olive oils are freshly produced or are stored for a period of time, since the storage of olive oil during period leads to the loss in the quality of olive oils. The second aim is to evaluate the effectiveness of the chemometric classification tools that we have used for the determination and prediction of the olive oil category.

## 2. Materials and Methods

### 2.1. Sampling

This study was carried out on 81 samples of monovarietal (Picholine) virgin olive oil from Morocco. These oils were stored in the dark at a temperature range of 10 ± 1°C. To preserve the molecular qualities of olive oils for a shelf life of 0 and 24 months, as shown in Table 1. During the storage period, the olive oil did not undergo any freezing.

To carry out this study, 63 samples were used for calibration and 18 for external validation of the models built.

### 2.2. Spectral Fluorescence Acquisition

The fresh and stored virgin olive oils are directly analyzed by fluorescence spectroscopy, using the FluoroMax-4 (Jobin Yvon) spectrophotometer. These fluorescence measurements are carried out using a fluorescence cuvette with polytetrafluoroethylene (PTFE) cover, UV quartz with a light path of 10 mm.

The acquisition of emission spectra of olive oil has been made at an excitation wavelength of 400 nm and emission wavelength which ranged from 415 nm to 785 nm with a step of 0.5 nm. Some fluorescent molecules of the olive oil have been excited following the absorption of photons at this wavelength which allows them to enter into an electronically excited state; these molecules will return to their fundamental state by emitting photons with a wavelength greater than the excitation wavelength.

### 2.3. Multivariate Data Analysis

Multivariate data analysis is a group of statistical methods that focus on the simultaneous observation, exploitation, and processing of several statistical variables in order to extract relevant synthetic information. These chemometric tools are generally divided into two groups, unsupervised methods such as PCA and supervised methods such as PLS-DA, LDA, and SVM. Generally, these supervised methods are part of the discriminant analysis that consists in determining the belonging of an individual to a predefined group according to the observation of predictive qualitative variables. These discriminant analyses can provide additional details on the obtained results, such as the identification of the variables that leads to the creation of the typology groups. The visualization of the results of this analysis can take the form of a mapping similar to the PCA score plot, where the different individuals are grouped together according to their group affiliation.

Principal component analysis (PCA) is an extremely powerful unsupervised method of synthesizing information, very useful when there is a large amount of quantitative data to be processed and interpreted. As a basic tool in chemometrics, PCA serves different purposes: exploration and description of a dataset, preparation and cleaning of data, identification of individual groups, and preliminary step for another chemometric treatment, LDA and SVM.

The supervised partial least squares discriminant analysis (PLS-DA) is the use of the PLS2 regression method, where the response variable is a categorical variable expressing the membership class of the units. This response is coded to contain only two whole numbers. In general, 0 and 1 are used to indicate “outside the group” and “within the group,” respectively [33]. The components of this method are constructed by trying to find an adequate compromise between two main purposes: to describe the whole set of explanatory variables and to predict the response variables [34].

Linear discriminant analysis (LDA) is one of the most important methods of discrimination, and it consists in finding linear combinations of the discriminating variables, making it possible to discriminate the most compact and distant groups by using hyperplanes. In the case of spectral data, this method was often preceded by selections of variables because the model produced is often difficult to interpret in the absence of initial variable selections, and the results are unstable in the case of correlations between variables, as it is always the case with spectral data [35].

Support vector machine (SVM) is a method that belongs to the family of automatic learning algorithms that solve both classification and regression problems. However, it is commonly applied in classification objectives. It consists of finding *n*-hyperplane with the maximum margin distance between the points through the use of techniques called kernel trick. The most used algorithms are linear kernel, polynomial kernel, radial basis function kernel, and sigmoid kernel [36].

### 2.4. Software

All data processing of fluorescence spectra and applications of chemometric methods, principal component analysis, partial least squares discriminant analysis, support vector machine, and linear discriminant analysis have realized thanks to the Unscrambler software, version 10.4 camo analytic.

## 3. Results and Discussion

**Figure 1:**
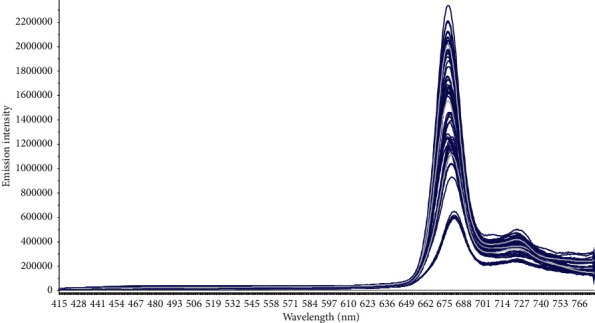
Fluorescence emission spectrum of fresh virgin olive oil and stored olive oil.

Figure 1 shows the emission spectra of fresh olive oils produced and of stored olive oils as shown in Table 1; these spectra present differences in the spectral intensity of the band corresponding to the maximum absorption at 675 nm. This band corresponds notably to the emission of some fluorescent molecules in olive oil; chlorophyll, and pheophytin [30], and these molecules are responsible for the green coloration of the olive oil and represent an important parameter of olive oil's quality.

**Figure 2:**
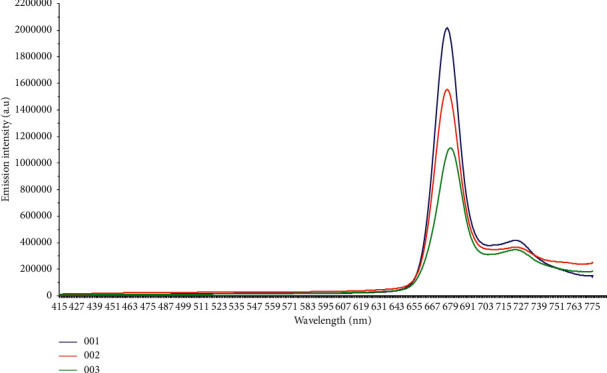
Average emission spectra of each group of olive oil (OO1 = fresh olive oil, OO2 = stored olive oil during 12 months, and OO3 = stored olive oil during 24 months).

The fluorescence spectra show that there is a decrease of the spectral intensity during the storage time due to the degradation of chlorophyll [37]. In fact, the spectra also show that the behavior of these oils is varying because the samples belong to different origins as geographical areas and mills. Consequently, different contents of chlorophyll pigments [7]. The average spectrum representation of each group of oil allows representing the behavior of the oils during the storage time as shown in Figure 2.

### 3.1. Principal Component Analysis (PCA)

**Figure 3:**
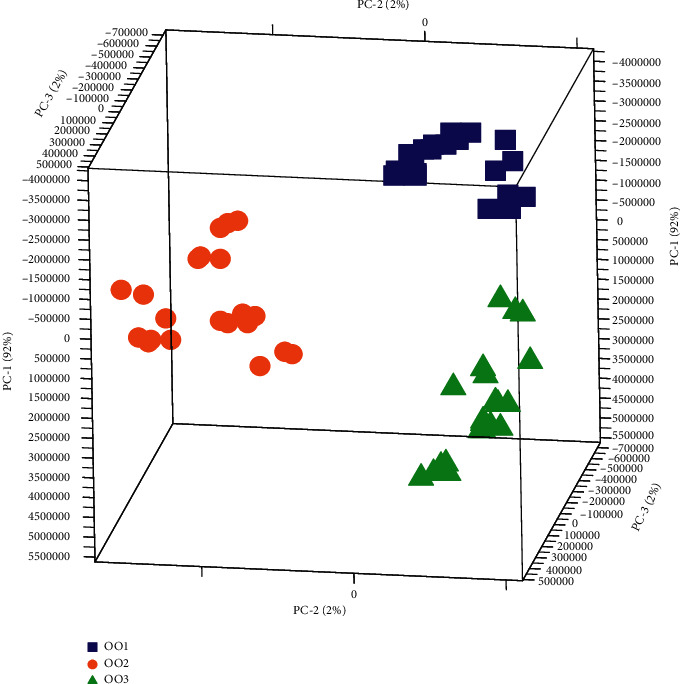
PCA 3D score plot of the first three principal components PC1-PC2-PC3 (OO1 = fresh olive oil, OO2 = stored olive oil during 12 months, and OO3 = stored olive oil during 24 months).

To describe the data in a very small dimensional space, a PCA has been firstly performed on the 81 spectra of olive oils to exploit the dataset and get pieces information on the distribution and the behavior of the samples concerning the measured variables that represent the wavelengths of the fluorescence spectral data.  Figure 3 illustrates the PCA 3D score plot.

**Figure 4:**
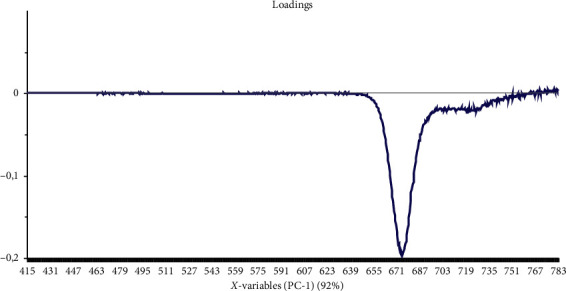
PCA loading plot of the first principal component PC1.

PCA shows that the first three principal components explain 96% of the total variability in the data: 92% for the first component and 4% for other components. Moreover, PCA shows that there is discrimination between the three groups of oil according to storage time; it also shows that there is an intragroup variability for each group. This classification is ensured essentially by the first component which represents the majority of the spectral information. The study of the loading (Figure 4) associated with the first PC shows that all weights are negative, which is the characteristic of chemical or biochemical effects on the spectra and not of physical characterization of the spectra. This remark allows us to show that the first axis represents the chlorophyll pigment content.

The separation tendency of olive oils according to the storage time was evident on the 3D-score plot PC1-PC2-PC3, which demonstrated the capability to use PCA on fluorescence data to identify the freshness and the storage time of the virgin olive oils.

### 3.2. Partial Least Squares Discriminant Analysis (PLS-DA)

In order to develop a supervised classification method capable of classifying and authenticating virgin olive oils according to their shelf life, the PLS-DA discrimination model has been developed for the three olive oil groups on 63 calibration samples using the NIPALS algorithm. The performance of the constructed models was evaluated using the root-mean-square error of calibration (RMSEC), the root-mean-square error of cross-validation (RMSECV), and the root-mean-square error of prediction (RMSEP) obtained by external validation and the slope of the regression *R*^2^.

**Figure 5:**
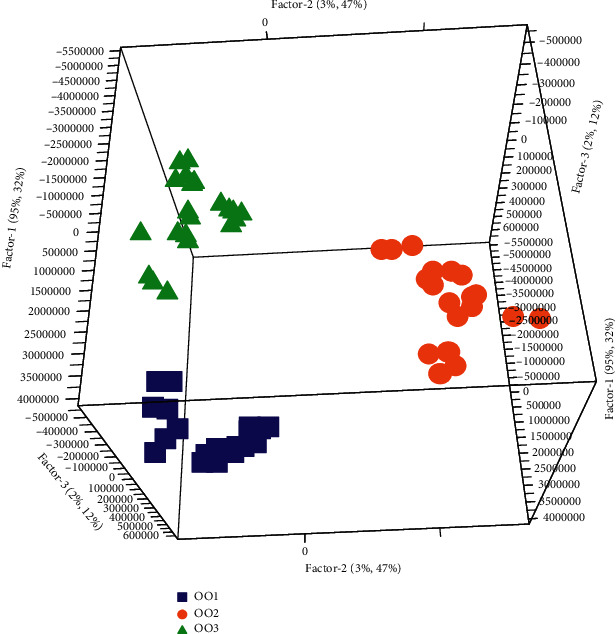
3D-PLS-DA score plots for the fluorescence spectra of olive oils groups (OO1 = fresh olive oil, OO2 = stored olive oil during 12 months, and OO3 = stored olive oil during 24 months).

The application of the discriminant PLS shows a high capability in the discrimination of the three groups of olive oils as shown in the score plot (Figure 5).

The discrimination quality of the constructed model is summarized in Table 2. The performance evaluation of the built models shows that the correlation coefficient ranges between 94% and 89% in the case of the calibration results and between 94% and 86% in the case of cross-validation results, while the mean square error of the calibration ranges between 0.11 and 0.16, and for the cross-validation, it ranges between 0.12 and 0.18.

The predictive performance of the constructed calibration models have been evaluated by external validation using external samples (6 samples of each class). The predicted *y*-value of a new sample near to 1 (or greater than 0.5) allocates the sample to a specific category, while a sample with a predicted *y*-value less than 0.5 is allocated outside the category [33].

The results of external sample prediction by the constructed models mentioned Table 3 show that these samples have been clearly assigned to their respective classes with a perfect accuracy of 100%.

### 3.3. Support Vector Machine Classification (SVM)

SVM (type C-SVC) has been applied on the fluorescence spectral data of the three groups of olive oils, using a linear kernel algorithm. The reported results in Table 4 show that the model has provided a good classification performance for the three classes of oils according to their membership (freshness and storage time). The calibration model has been validated using firstly cross-validation that shows a significant accuracy of classification that reached 100%. Finally, we used an external validation by a new set of samples (6 samples of each class) to evaluate the predictive performance of the constructed model. The 18 samples of the sample set have clearly been attributed to their respective classes with a perfect accuracy of 100%. The results that we have obtained by the SVM model confirm the predictive capability to classify the different classes of samples according to their freshness and storage time.

### 3.4. Linear Discriminant Analysis (LDA)

The supervised discrimination method was also used; LDA has been applied on the three synthetic variables generated by the PCA. This method is not applicable on the data where the variables have colinearity among themselves; for this reason, it is necessary to combine this method with methods of variable selection like the PCA method, because the PCA allows to generate independent synthetic variables from the initial variables. The application of the LDA method on the first three components of the PCA shows a very high capacity of discrimination between the three classes of olive oil as shown in Table 5. This classification model provides a high discrimination performance of the three classes according to their membership. The results of calibration and cross-validation show that this model can correctly classify the three classes with an accuracy that reaches 100%.

The predictive assessment of this model was done through external validation by a new set of samples (6 samples of each category). The 18 samples of the test set are clearly assigned to their respective categories ensuring a perfect accuracy of 100% as reported in Table 5.

It is clear that the ideal situation occurs when all VOO samples arrive at the diagonal cells of the matrix. That is to say, each olive oil class was correctly classified by the SVM, PLS-DA, and ACP-LDA models, which led to a 100% success rate in the classification of the three Moroccan oil groups according to their freshness. This success rate was also higher than that of Sinelli et al. [38], who found 87% by combining physicochemical data (acidity (%), peroxide value, and K_232_ and K_270_) with linear discriminant analysis and 98% by using mid-infrared spectroscopy.

The improvement of this method with a wide range of olive oils by the introduction of several varieties of olive oil of different freshness allows to increase the analytical performance of this method and to use it as a routine method for the authentication of the freshness of olive oils in analytical laboratories. Such a process allows many control authorities to check the freshness of olive oils on the market in order to protect the consumer against fraudulent actions.

## 4. Conclusion

The present study shows the capability of fluorescence spectroscopy coupled to supervised and unsupervised methods for the classification and the prediction of freshly produced virgin olive oils and virgin olive oils that have been stored during a time.

The obtained results by PCA as an unsupervised method of exploitation and grouping of individuals show that there is discrimination between the three groups of olive oils concerning the variables measured by fluorescence spectroscopy.

The application of the supervised classification methods, PLS-DA, SVM, and LDA, shows a very high capacity in the discrimination between these three categories of oil. They also show a very accurate capacity for the prediction and correct classification of external samples in its class.

For a reliable process of rapid evaluation and authentication of virgin olive oils in the market to identify the freshness of olive oils, the development of robust spectral databases is encouraged as much as possible.

## Figures and Tables

**Table 1:** Storage conditions for virgin olive oil.

Number of samples	Origin	Type of mills	Variety	Light condition	Temperature condition	Storage time (months)
3	Beni Mellal province	Traditional mill	Picholine	Darkness	10 ± 1	0
4		Modern mill	Picholine	Darkness	10 ± 1	0
7	Khenifra province	Modern mill	Picholine	Darkness	10 ± 1	0
2	Khouribga province	Traditional mill	Picholine	Darkness	10 ± 1	0
5		Modern mill	Picholine	Darkness	10 ± 1	0
6	Fquih Ben Salah province	Modern mill	Picholine	Darkness	10 ± 1	0
3	Beni Mellal province	Traditional mill	Picholine	Darkness	10 ± 1	12
4		Modern mill	Picholine	Darkness	10 ± 1	12
7	Khenifra province	Modern mill	Picholine	Darkness	10 ± 1	12
2	Khouribga province	Traditional mill	Picholine	Darkness	10 ± 1	12
5		Modern mill	Picholine	Darkness	10 ± 1	12
6	Fquih Ben Salah province	Modern mill	Picholine	Darkness	10 ± 1	12
3	Beni Mellal province	Traditional mill	Picholine	Darkness	10 ± 1	24
4		Modern mill	Picholine	Darkness	10 ± 1	24
7	Khenifra province	Modern mill	Picholine	Darkness	10 ± 1	24
2	Khouribga province	Traditional mill	Picholine	Darkness	10 ± 1	24
5		Modern mill	Picholine	Darkness	10 ± 1	24
6	Fquih Ben Salah province	Modern mill	Picholine	Darkness	10 ± 1	24

**Table 2:** Statistical parameters of the built models with and without data preprocessing (PLS-DA).

Label	Preprocessing	Number of latent variable	Calibration		Cross validation	
			*R*-square (%)	RMSEC	*R*-square (%)	RMSECV
OO1	Without preprocessing	3 LV	91	0.14	89	0.16
OO2			94	0.12	93	0.13
OO3			89	0.16	89	0.17
OO1	Smoothing (Savitzky and Golay)	3 LV	90	0.15	89	0.16
OO2			94	0.12	94	0.12
OO3			89	0.16	87	0.17
OO1	Detrend (polynomial 1)	3 LV	91	0.15	86	0.18
OO2			95	0.11	90	0.15
OO3			89	0.16	87	0.17

**Table 3:** External validation of the classification of PLS-DA models for the fluorescence spectra of the three categories of olive oil.

Confusion matrix	Label	OO1	OO2	OO3	Accuracy of external validation (%)
Predicted external set	OO1	6	0	0	100
	OO2	0	6	0	
	OO3	0	0	6	

**Table 4:** Confusion matrix for the classification of training and external dataset using the SVM method.

Confusion matrix	Actual				Accuracy	
	Label	OO1	OO2	OO3	Calibration	Cross-validation
Predicted training set	OO1	21	0	0	100%	100%
	OO2	0	21	0		
	OO3	0	0	21		

		OO1	OO2	OO3	External validation	

Predicted external set	OO1	6	0	0	100%	
	OO2	0	6	0		
	OO3	0	0	6		

**Table 5:** Confusion matrix for the classification of training and external dataset using the PCA-LDA method.

Confusion matrix	Actual				Accuracy	
	Label	OO1	OO2	OO3	Calibration	Cross-validation
Predicted training set	OO1	21	0	0	100%	100%
	OO2	0	21	0		
	OO3	0	0	21		

		OO1	OO2	OO3	External validation	

Predicted external set	OO1	6	0	0	100%	
	OO2	0	6	0		
	OO3	0	0	6		

## Data Availability

The data used to establish this research are available on request from the corresponding author.
